# Prevalence and risk factors of gestational diabetes mellitus in Asia: a systematic review and meta-analysis

**DOI:** 10.1186/s12884-018-2131-4

**Published:** 2018-12-14

**Authors:** Kai Wei Lee, Siew Mooi Ching, Vasudevan Ramachandran, Anne Yee, Fan Kee Hoo, Yook Chin Chia, Wan Aliaa Wan Sulaiman, Subapriya Suppiah, Mohd Hazmi Mohamed, Sajesh K. Veettil

**Affiliations:** 1 Department of Family Medicine, Faculty of Medicine and Health Sciences, Universiti Putra Malaysia, 43400 Serdang, Malaysia; 2 Malaysian Research Institute on Ageing, Universiti Putra Malaysia, 43400 Serdang, Malaysia; 3 Department of Psychological Medicine, University of Malaya Center for Addiction Sciences (UMCAS), Faculty of Medicine, University of Malaya, 50603 Kuala Lumpur, Malaysia; 4 Department of Medicine, Faculty of Medicine and Health Sciences, Universiti Putra Malaysia, 43400 Serdang, Malaysia; 5 Department of Medical Sciences, School of Healthcare and Medical Sciences, Sunway University, 47500 Bandar Sunway, Selangor Malaysia; 6 Department of Imaging, Faculty of Medicine and Health Sciences, Universiti Putra Malaysia, 43400 Serdang, Malaysia; 7 Department of Surgery, Faculty of Medicine and Health Sciences, Universiti Putra Malaysia, 43400 Serdang, Malaysia; 8 Department of Pharmacy Practice, School of Pharmacy, International Medical University, 57000 Kuala Lumpur, Malaysia

**Keywords:** Prevalence, Risk factors, Gestational diabetes mellitus, Asia; meta-analysis

## Abstract

**Background:**

Gestational diabetes mellitus (GDM) is a of the major public health issues in Asia. The present study aimed to determine the prevalence of, and risk factors for GDM in Asia via a systematic review and meta-analysis.

**Methods:**

We systematically searched PubMed, Ovid, Scopus and ScienceDirect for observational studies in Asia from inception to August 2017. We selected cross sectional studies reporting the prevalence and risk factors for GDM. A random effects model was used to estimate the pooled prevalence of GDM and odds ratio (OR) with 95% confidence interval (CI).

**Results:**

Eighty-four studies with STROBE score ≥ 14 were included in our analysis. The pooled prevalence of GDM in Asia was 11.5% (95% CI 10.9–12.1). There was considerable heterogeneity (I^2^ > 95%) in the prevalence of GDM in Asia, which is likely due to differences in diagnostic criteria, screening methods and study setting. Meta-analysis demonstrated that the risk factors of GDM include history of previous GDM (OR 8.42, 95% CI 5.35–13.23); macrosomia (OR 4.41, 95% CI 3.09–6.31); and congenital anomalies (OR 4.25, 95% CI 1.52–11.88). Other risk factors include a BMI ≥25 kg/m^2^ (OR 3.27, 95% CI 2.81–3.80); pregnancy-induced hypertension (OR 3.20, 95% CI 2.19–4.68); family history of diabetes (OR 2.77, 2.22–3.47); history of stillbirth (OR 2.39, 95% CI 1.68–3.40); polycystic ovary syndrome (OR 2.33, 95% CI1.72–3.17); history of abortion (OR 2.25, 95% CI 1.54–3.29); age ≥ 25 (OR 2.17, 95% CI 1.96–2.41); multiparity ≥2 (OR 1.37, 95% CI 1.24–1.52); and history of preterm delivery (OR 1.93, 95% CI 1.21–3.07).

**Conclusion:**

We found a high prevalence of GDM among the Asian population. Asian women with common risk factors especially among those with history of previous GDM, congenital anomalies or macrosomia should receive additional attention from physician as high-risk cases for GDM in pregnancy.

**Trial registration:**

PROSPERO (2017: CRD42017070104).

## Background

Gestational diabetes mellitus (GDM) is defined as any degree of dysglycaemia that occurs for the first time or is first detected during pregnancy [1, 2]. It has become a global public health burden [3]. GDM is one of the leading causes of mortality and morbidity for both the mother and the infant worldwide [4–13]. Mothers with GDM are at risk of developing gestational hypertension, preeclampsia and caesarean section [7, 14–16]. Apart from this, women with a history of GDM are also at significantly higher risk of developing subsequent type 2 diabetes mellitus (T2DM) and cardiovascular diseases [17, 18]. Babies born from GDM women are at risk of being macrosomic, may suffer from more congenital abnormalities and have a greater propensity of developing neonatal hypoglycaemia, and T2DM later in life [7, 19–24]. As such, it is important for healthcare policy makers to understand the burden of GDM for early detection and further intervention.

Up to now, there has been no gold standard criterion for the diagnosis. Different countries use different diagnostic criteria in determining its prevalence (Appendix 1). Based on these criteria, the estimated prevalence of GDM worldwide is 7.0% [25]. Prevalence varies from 5.4% in Europe [26] to 14.0% Africa [27]. In Asia, the prevalence of GDM ranges from 0.7 to 51.0% [28–30]. This vast disparity in prevalence rates may be due to differences in ethnicity [28, 30], diagnostic criteria [31–33], screening strategies [29, 34], and population characteristics [35, 36].

Diagnostic criteria have been developed by numerous associations such as: O′ Sullivan; American Diabetes Association (ADA); Australian Diabetes in Pregnancy Society (ADIPS); Carpenter-Coustan (CC); International Association of the Diabetes and Pregnancy Study Groups (IADPSG); International Classification of Diseases (ICD); European Association for the Study of Diabetes (EASD); The American College of Obstetricians and Gynecologists (ACOG); Diabetes in Pregnancy Study group of India (DIPSI); Japan Diabetes Society (JDS); National Diabetes Data Group (NDDG); and World Health Organization (WHO); Canadian Diabetes Association (CDA); and so on. These diagnostic criteria vary in terms of screening methods and screening threshold.

Diagnosis of GDM primarily depends on the results of an oral glucose tolerance test (OGTT). The OGTT can be carried out via a 75-g two-hour test or a 100-g three-hour OGTT. The 75-g two-hour OGTT is a one-step approach, while the 100-g three-hour OGTT is usually implemented as the second step of a two-step approach. A diagnosis of GDM is made when one glucose value is elevated for the 75-g two-hour OGTT. Despite the presence of multiple diagnostic criteria to diagnose GDM, to date, there has been a degree of uncertainty around the optimum thresholds for a positive test [25, 37–59]. The thresholds for an elevated fasting glucose range from 92 mg/dl (5.1 mmol/L) to 140 mg/dl (7.8 mmol/L) [41, 44] while values for the two hours after OGTT range from 7.8 to 11.1 mmol/L [44, 46]. The IADPSG criteria is the most commonly used threshold for defining elevated values recently following the Hyperglycemia and Adverse Pregnancy Outcome (HAPO) study [60]. Overall, the 75-g two-hour test is more practical and convenient compared with the 100-g three-hour test. Furthermore, it appears to be more sensitive in predicting the pregnancy’s complication like gestational hypertension, preeclampsia and macrosomia than the 100-g three-hour test [61]. The reason for increased sensitivity is mainly that only one elevated glucose value is needed to diagnose GDM in 75-g two-hour test compared to 100-g three-hour test which requires two abnormal glucose values [60]. The thresholds used to define the abnormal values in the 100-g three-hour test have been based on the Carpenter and Coustan, NDDG and O’Sullivan criteria [49–51].

Moreover, the prevalence of GDM is expected to increase over years [62–64], especially in Asia. This is possibly due to increase in maternal age and obesity in Asia [65, 66]. A recent review reported the prevalence of GDM in Eastern and Southeast Asia is 10.1% (95% CI: 6.5–15.7%) [29]. There has been no review on the overall prevalence of GDM in Asia. Therefore, the aim of this meta-analysis is to estimate the prevalence of GDM in a broader scope including the countries across Asia. In addition, we also examine the odds ratio of risk factors for GDM among the Asian populations.

The recognition of risk factors of GDM for the Asian population is therefore important to identify women at risk, making an early diagnosis and instituting intensive lifestyle modification and metformin treatment to control blood glucose to reduce the likelihood of problems of GDM, before they become more severe. This may help prevent or ameliorate adverse complications.

We therefore conducted a systematic review and meta-analysis to determine the prevalence and factors associated with GDM in Asia.

## Methods

The present review was registered with PROSPERO (2017: CRD42017070104) and conducted according to the Preferred Reporting Items for Systematic Reviews and Meta-Analyses (PRISMA) [67].

### Search strategy

Four databases were searched (PubMed, Ovid, Scopus and ScienceDirect) to do the literature search with the following search terms: (prevalence or incidence and/or risk factor) and (gestational diabetes or diabetes in pregnancy or gestational diabetes mellitus) and (Asia). A combination of expanded MeSH term and free-text searches were used as shown in Appendix 2. Then the reference lists of relevant articles were screened for its suitability to be recruited into this review.

### Inclusion criteria

Any studies in Asia that reported prevalence and risk factors for GDM and fulfilled the following criteria were entered into the analysis, including the following factors: (1) conducted in Asian countries classified by the United Nations Statistics Division [68]; (2) reported prevalence and risk factors as primary results; (3) English peer review articles published in journals from inception to August 22, 2017; and (4) a sample size no less than 100 subjects. When several publications were actually derived from the same dataset or cohorts, we chose the data from the latest publication or largest cohort only. Similarly, when different screening criteria was used to diagnose GDM, we used the criteria with the highest prevalence for the risk factor calculation. We identified other pertinent studies through reverse-forward citation tracking and reference lists of related review articles.

### Study selection

We imported those relevant articles identified through the databases into EndNote programme X5 version and we removed duplicate publications. Two reviewers independently performed the screening using the titles and abstracts to search for potentially eligible articles based on the inclusion and exclusion criteria mentioned above. If there was a lack of information on the prevalence of GDM in the title and/or abstract, the full text was retrieved for further assessment. Discussions were held to resolve any disagreement for a final consensus before reviewing the full text each relevant article.

### Quality assessment and data extraction

The checklist Strengthening the Reporting of Observational Studies in Epidemiology (STROBE) was used to assess the quality of searched articles by two independent investigators [69]. The tool consists of 22 items that assess components in observation studies and whenever the information provided was not enough to assist in making judgement for a certain item, we agreed to grade that item with a ‘0’ meaning high risk of bias. Each article’s quality was graded as ‘good’ if STROBE score ≥14/22; or graded as ‘poor’ if STROBE score < 14/22 [69]. In this review, studies with STROBE score ≥ 14 were included in analysis. The scoring result was shown in Appendix 3.

One of the reviewers recorded the data from the selected studies into the extraction form using Excel, while the second reviewer verified the accuracy and completeness of the extracted data. The characteristics of the selected studies were extracted as follows: first author, year of publication, year of survey, country, setting, gestational age, screening procedure (one and/or two steps), diagnostic criteria for GDM, sample size, GDM cases, prevalence of GDM, odds ratio, relative risk of certain risk factors. Since we only collected published studies, the outcome measures extracted were gestational diabetes incidence and risk factors in terms of differences of proportion/percent of gestational diabetes in the total subjects examined. No ethics approval was needed in this review as the work consisted of secondary data collection and analysis only.

### Data analysis

A random-effects (DerSimonian and Laird method) meta-analysis was used to pool the prevalence and odds ratio (OR) estimated from individual studies and reported with 95% confidence interval (CI). Heterogeneity across studies was assessed using the I^2^ index (low is < 25%, moderate 25–50%, and high > 50%), indicating the percent of total discrepancy due to studies variation [70]. Subgroup analyses for prevalence were performed by country, diagnostics criteria, screening methods and study setting. For Statistical analysis, StatDirect Statistical Software version 2.7.9 was employed.

The prevalence of GDM in Asia was analysed by subgrouping the country, and by the 10 different diagnostic criteria according to (1) IADPSG, (2) China Ministry of Health (China MOH), (3) ADA, (4) WHO, (5) DIPSI, (6) CC, (7) NDDG, (8) CC and WHO, (9) ICD 10th (ICD-10), (10) JDS. The data were also analysed by subgrouping the screening method and study setting.

The risk factors for GDM were reported in odds ratio (OR) with 95% confidence interval (CI) by using a random effect.

### Operational definitions

Oral glucose tolerance test (OGTT) is a diagnostic test for gestational diabetes mellitus based on the glucose concentration in venous plasma using an accurate and precise enzymatic method [71]. Congenital anomaly in infants was defined as malformations involving the cardiovascular, genitourinary, musculoskeletal, and central nervous systems [72].

## Results

### Description of included studies

**Fig. 1:**
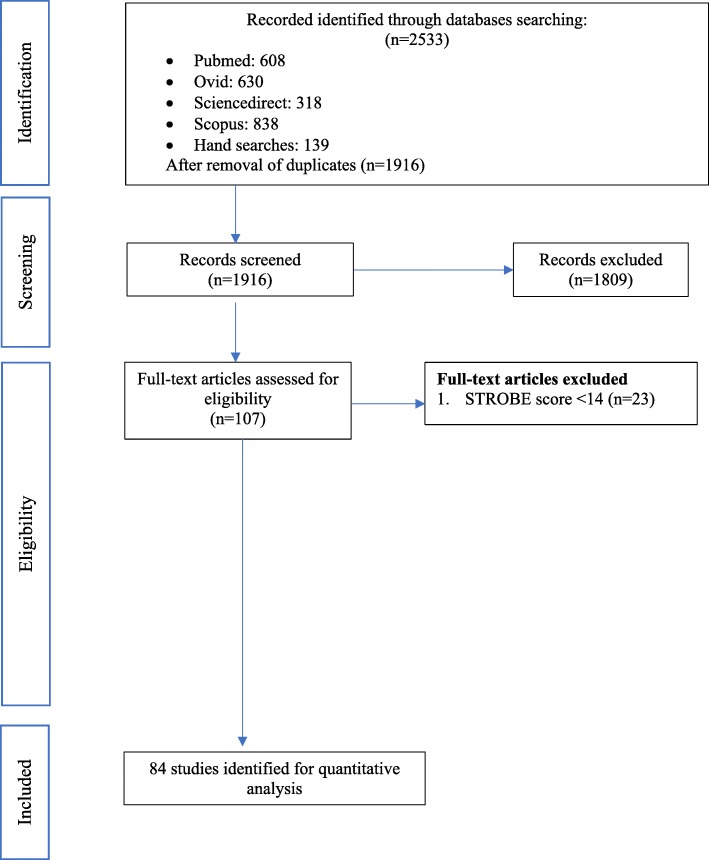
PRISMA flow diagram of the literature screening process

We identified 2533 manuscripts in the initial search as shown in Fig. 1. After removal of duplicate records (*n* = 617), 1916 studies were retrieved for further assessment. After careful evaluation of the inclusion/exclusion criteria, 107 studies fulfilled our criteria. Among 107 studies, 84 studies (1988–2017) were of STROBE score of ≥14. These studies were and these studies were included in this systematic review and meta-analysis.

### Characteristics of included studies

The main characteristics of the included studies are shown in the Appendix 4. A total sample of 2, 314,763 pregnant women from 20 countries were included in the analysis. Twenty-four were in India [73–96], nine in Iran [97–105], 8 in China [106–113], 7 in Saudi Arabia [28, 114–119], four in Thailand [120–123], Sri Lanka [124–127] and Japan [128–131], three in South Korea [132–134], Bangladesh [135–137] and Israel [138–140]. Additionally, two were in Vietnam [141, 142], Malaysia [143, 144], Qatar [145, 146], Pakistan [147, 148] and Nepal [149, 150]. One each were from Yemen [151], Hong Kong [152], Singapore [153], Taiwan [154] and Turkmenistan [155].

In terms of diagnostic criteria, a total of 23 studies used the WHO criteria, 13 used IADPSG, 13 used ADA, 13 used CC, 12 used DIPSI, 4 used NDDG, 3 used JDS, 1 used ICD-10, 1 used China MOH criteria and 1 used the combination of the CC and WHO criteria (Table 1). Out of 84 studies, the most commonly used one-step screening procedure was applied in 53 studies (Table 1). A One step screening procedure is defined as the pregnant women undergoing a 75 g OGTT. Two-step screening procedure was used in 30 studies. Two-step screening procedure is defined as pregnant women firstly undergoing a 50 g one-hour Glucose Challenge Test (GCT). If the woman tested positive in the 50 g GCT, they were then required to undergo either a 75 g or 100 g OGTT.

**Table 1:** Pooled prevalence and 95% confidence interval of gestational diabetes by subgroup analysis

Variable	*N*	Total sample size	Total GDM	Prevalence, %	95% CI	*P*-value	I^2^, %
Country							
Taiwan	1	132	51	38.6	30.3–46.9	NA	NA
Hong Kong	1	520	169	32.5	28.5–36.5	NA	NA
Saudi Arabia	7	13,865	3192	22.9	12.9–32.9	99.51	< 0.0001
Vietnam	2	5474	1224	22.3	18.4–26.2	91.94	< 0.0001
Malaysia	2	2136	359	18.5	6.2–30.8	97.97	< 0.0001
Singapore	1	909	160	17.6	15.1–20.1	NA	NA
Thailand	4	24,168	1872	17.1	6.3–27.8	99.11	< 0.0001
Iran	9	9872	1146	14.9	10.2–19.6	98.58	< 0.0001
Qatar	2	2205	323	13.3	7.4–19.3	93.54	< 0.0001
China	8	156,942	11,394	12.6	8.6–16.7	99.78	< 0.0001
Sri lanka	4	3577	380	11.4	5.1–17.8	97.93	< 0.0001
South Korea	3	1,316,307	98,845	10.5	5.8–15.3	99.87	< 0.0001
India	24	17,049	1679	8.8	6.7–10.9	96.57	< 0.0001
Bangladesh	3	2785	226	8.2	6 l.0–10.5	71.61	0.03
Pakistan	2	1642	127	7.7	6.4–9.0	0	0.752
Turkmenistan	1	1620	109	6.7	5.5–7.9	NA	NA
Israel	3	737,978	36,822	5.3	3.7–7.0	99.89	< 0.0001
Yemen	1	311	16	5.1	2.7–6.6	NA	NA
Japan	4	15,109	390	2.8	1.9–3.7	84.4	< 0.0001
Nepal	2	2162	26	1.5	0.2–3.2	84.13	0.012
Subtotal	84	2,314,763	158,510	11.5	10.9–12.1	99.57	< 0.0001
Diagnostic criteria							
IADPSG	13	42,317	5148	20.9	17.3–24.6	99.17	< 0.0001
CHINA MOH	1	14,986	2987	19.9	19.3–20.6	NA	NA
ADA	13	379,583	15,501	13.9	11.5–16.2	98.68	< 0.0001
WHO	23	134,152	9750	13	9.6–16.4	99.38	< 0.0001
DIPSI	12	9879	1114	8.3	5.7–10.9	94.76	< 0.0001
CC	13	384,146	23,714	7.6	6.6–8.7	99	< 0.0001
NDDG	4	31,734	1577	4.3	1.4–7.3	99.2	< 0.0001
CC&WHO	1	2000	75	3.7	2.9–4.6	NA	NA
ICD-10	1	1,306,281	98,403	3.7	1.2–6.2	NA	NA
JDS	3	9685	241	3.6	1.2–6.0	88.33	< 0.0001
Subtotal	84	2,314,763	158,510	11.5	10.9–12.1	99.59	< 0.0001
Setting							
Hospital	71	423,878	31,598	12.1	11–13.1	99.34	< 0.0001
Community	13	1,890,885	126,912	11.1	9.8–12.5	99.87	< 0.0001
Subgroup	84	2,314,763	158,510	11.5	10.9–12.1	99.59	< 0.0001
Screening Methods							
One-step	53	631,808	38,515	14.7	13.5–15.9	99.5	< 0.0001
Not stated	1	1,306,281	98,403	7.5	7.5–7.6	NA	NA
Two-steps	30	376,674	21,592	7.2	6.4–8.0	98.82	< 0.0001
Subtotal	84	2,314,763	158,510	11.5	10.9–12.1	99.57	< 0.0001

The setting of the study was examined in subgroup analysis; 71 studies were hospital-based and 13 studies were community based.

### Prevalence of GDM

**Fig. 2:**
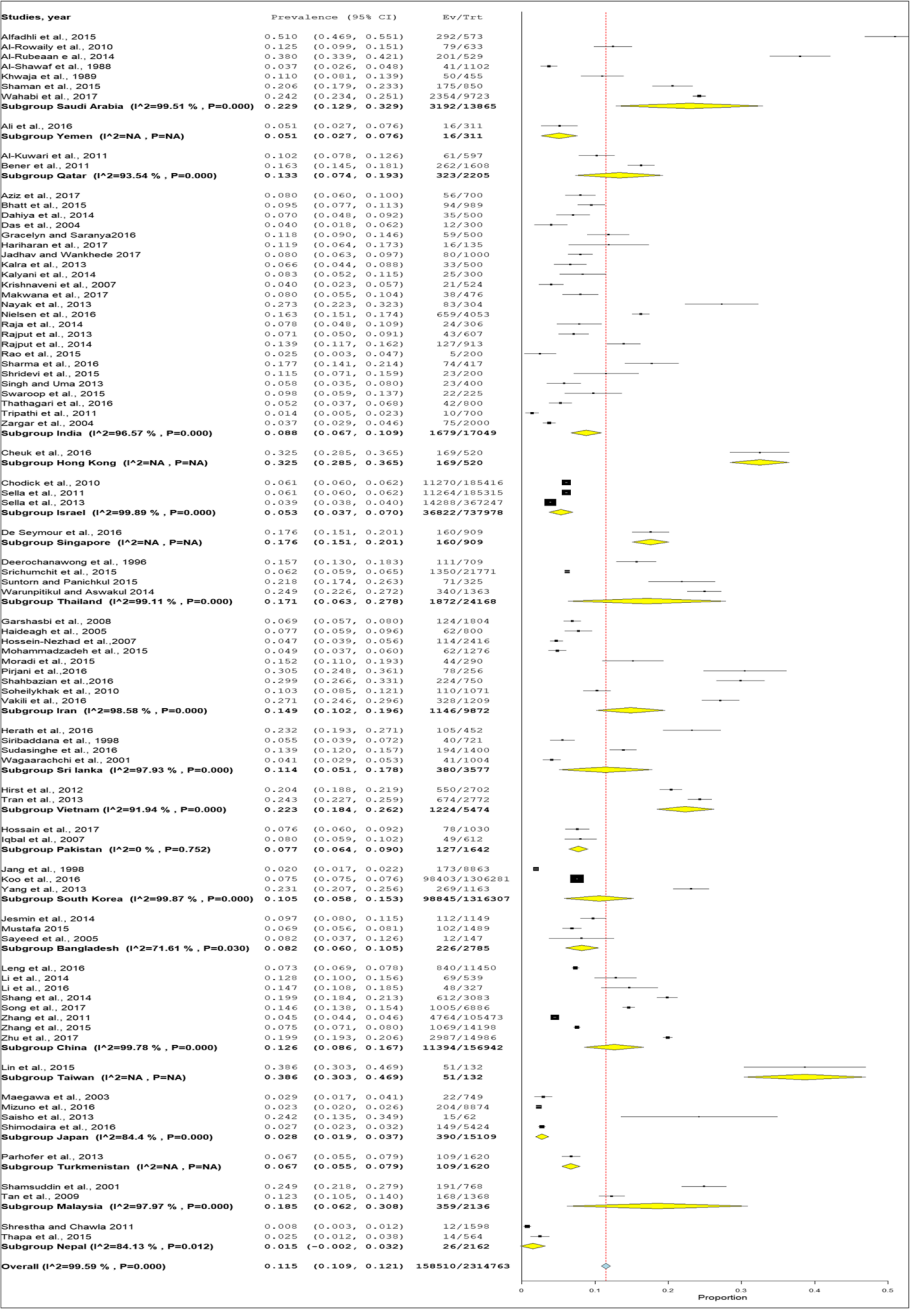
The forest plot of the prevalence of gestational diabetes mellitus in Asia

The overall mean prevalence of GDM was 11.5% (95% CI 10.9–12.1) (Fig. 2). Table 1 shows the prevalence of GDM across difference covariates such as by country, diagnostics criteria, screening step and study setting. The prevalence of GDM by country was highest in Taiwan (38.6%), followed by Hong Kong (32.5%) and Saudi Arabia (22.9%). The lowest prevalence of GDM was in Nepal (1.5%) followed by Japan (2.8%). The prevalence of GDM by diagnostic criteria was highest with IADPSG (20.9%) followed by China MOH (19.9%). The prevalence of GDM was much lower when the studies used the common and popular criteria of WHO 1980–2013 or ADA 2002–2014 (13.0 to 13.9%) versus the IADPSG and China MOH which gave a prevalence of 19.9 and 20.9%, respectively. The prevalence of GDM by screening methods was very different, where the one-step screening methods reported a prevalence of GDM of 14.7%, while the prevalence of GDM two-step screening method (7.2%) was half that of the one-step method. The prevalence of GDM was almost similar between hospital and community setting (12.1% versus 11.1%).

### Risk factors of GDM

The risk factors of GDM was analysed in this current review. The most important risk factors in GDM among Asian population were rated based on pooled analysis of the included studies (Table 2). This meta-analysis found that the odds of GDM was increased by history of previous GDM (OR 8.42, 95% CI: 5.35–13.23), congenital anomalies (OR 4.25, 95% CI 1.52–11.88), and macrosomia (OR 4.41, 95% CI 3.09–6.31). Other risk factor included BMI ≥25 (OR 3.27, 95% CI 2.81–3.80) and pregnancy-induced hypertension (PIH) (OR 3.20, 95% CI 2.19–4.68).

**Table 2:** Pooled prevalence and 95% confidence interval of gestational diabetes according to the risk factors

Variable	*N*	Exposure in GDM	Total GDM	Exposure in Non-GDM	Total Non-GDM	OR	95% CI	I^2^, %	*P*-value
History of previous GDM	24	343	3246	272	20,646	8.42	5.35–13.23	80.92	< 0.001
History of congenital anomalies	6	32	655	50	3262	4.25	1.52–11.88	64.64	0.015
History of macrosomia	29	397	4275	1001	29,506	4.41	3.09–6.31	81.14	< 0.001
BMI ≥ 25 kg/m^2^	33	13,304	42,306	80,126	582,707	3.27	2.81–3.80	93.49	< 0.001
PIH	12	163	1891	612	18,468	3.2	2.19–4.68	68.96	< 0.001
Family History of Diabetes	60	3177	11,068	12,336	94,962	2.77	2.22–3.47	93.76	< 0.001
History of stillbirth	25	261	2786	1158	21,257	2.39	1.68–3.40	75.38	< 0.001
PCOS	7	2424	113,827	26,777	1,566,026	2.33	1.72–3.17	94.07	< 0.001
History of abortion	19	803	2658	2404	16,844	2.25	1.54–3.29	91.37	< 0.001
Age ≥ 25	34	226,788	354,080	2,637,545	4,798,678	2.17	1.96–2.41	96.91	< 0.001
Multiparity ≥2	32	21,069	31,901	290,125	434,198	1.37	1.34–1.52	86.55	< 0.001
History of preterm delivery	9	230	2274	837	12,748	1.93	1.21–3.07	76.09	< 0.001
History of neonatal death	5	26	550	58	1593	1.8	0.86–3.79	43.29	0.133
Illiteracy	7	118	2919	604	10,372	1.29	0.82–2.04	65.63	0.008
Current smoking	8	1257	14,162	18,924	213,495	1.04	0.98–1.11	0	0.93
Current drinking	5	30	2422	916	38,433	0.79	0.54–1.14	0	0.66
Primigravida	18	7363	8753	38,871	47,228	0.55	0.41–0.73	85.99	< 0.001

Risk factors such as family history of diabetes (OR 2.77, 2.22–3.47), history of stillbirth (OR 2.39, 95% CI 1.68–3.40), Polycystic ovary syndrome (PCOS) (OR 2.33, 95% CI1.72–3.17), history of abortion (OR 2.25, 95% CI 1.54–3.29), age ≥ 25 (OR 2.17, 95% CI 1.96–2.41), multiparity ≥2 (OR 1.37, 95% CI 1.24–1.52), and a history of preterm delivery (OR 1.93, 95% CI 1.21–3.07) in relation to GDM, ranging from 1.93–2.77 (*p* value < 0.05). On the other hand, for risk factors such as history of neonatal death, illiteracy and current smoking, the odds for GDM ranged from 1.04 to 1.80 (p value > 0.05). Primigravida status and current drinking was found to be protective factors for GDM with an OR of 0.55 and 0.79 (p value < 0.05), respectively.

## Discussion

The present meta-analysis included 84 studies from 20 countries across Asia. We compiled the prevalence and risk factors data from a huge population size (*n* = 2,314,763). The pooled prevalence of GDM was 11.5% (95% CI 10.9–12.1). This figure is considered more representative of the burden of GDM across Asian populations.

This prevalence of GDM in Asia is found to be higher than European countries (5.4%) but lower than in African countries (14.0%) [27, 51]. We have no clear reason for such a discrepancy, but we speculate that it may due to maternal age and BMI disparities, as well as ethnic background [156]. For example, South Asian have greater odds of developing GDM than White European and Black Africa at same age [157]. Similarly, South Asian women were older and more obese among GDM patients [157]. Therefore, advancing age, increasing BMI and racial group are associated to the high prevalence of GDM in Asia. It could also be due to a genetic predisposition of Asians to have a higher risk of insulin resistance compared to Caucasian [158]. The higher prevalence of GDM in Asia and Africa is higher than that of Europe. This is consistent with the higher prevalence of T2DM and GDM seen in Asia compared to Europe [62].

Prevalence of GDM including India and Middle Eastern countries makes a total of 20 countries. Our findings on prevalence of GDM are fairly similar to a recent study that reported the prevalence of GDM in 8 Eastern and Southeast Asian countries 10.1% (95% CI 6.5–15.7) [29].

The high heterogeneity in the overall prevalence seen in our study may be due to several reasons, such as different diagnostic criteria and screening methods used by different countries. For example, while several studies used the ADA criteria to screen for GDM, they also used different cut-off value of 92 mg/dl (5.1 mmol/l values as well) or 95 mg/dl (5.2 mmol/l) for the 75 g OGTT. Furthermore, even though within the same country, different diagnostic criteria were used to diagnose GDM. For example, seven diagnostic criteria were used in India and three in Vietnam, giving a broad range of prevalence of GDM ranging from 6.7–10.9 and 18.4.4–26.2, respectively. Hence it is not surprising that high heterogeneity of prevalence of GDM within a country is seen. Similarly, the sample size was important when determining prevalence of GDM, as the literature reports that there is a positive correlation between sample size and the prevalence [159]. In our meta-analysis, there were 5 studies [109, 133, 138–140] with a large sample size which gives larger weight to the prevalence of GDM. This may contributed to the heterogeneity in the results.

The IADPSG and China MOH diagnostic criteria usually results in higher prevalence of GDM where the prevalence can be higher by 3.5 to 45.3% [160]. This is partly because a lower cut-off value for fasting glucose is used [161]. These two diagnostic criteria are less popular in the screening for GDM. China MOH was another diagnostic criterion with higher prevalence of GDM. This criterion acknowledged hyperglycaemia in pregnancy be tested at an early stage of pregnancy and later divided them into T2DM in pregnancy and GDM [156] . Hence, this significantly increased the detection and prevalence rate.

The ADA and WHO criteria are the most popular diagnostic screening criteria used. The prevalence of GDM based on these criteria are lower than other criteria. There are also many different versions of these criteria over the years, with different cut-off glucose values to classify GDM. For instance, the WHO 2013 has a higher cut-off value for the 2-h plasma glucose compared to WHO 1999, and other diagnostic criteria. Different countries and studies used different diagnostic criteria and it has an impact on the prevalence of GDM. Using a lower threshold value in GDM screening would result in more cases compared to those using higher threshold values.

This review demonstrated differences in prevalence of GDM by subgroup screening methods in terms of other than diagnostic criteria that need to be examined when trying to explain the inconsistency in the prevalence of GDM between studies. In the analysis, the prevalence of GDM using one-step screening was nearly double that using the two-steps screening (14.7 and 7.2%. respectively).

This is an unexpected finding because a bigger dose of glucose of 75-g will be used in one-step screening method. In comparison with two step method, a 50-g oral glucose will be used in the first round so it will detect fewer GDM cases as only those who are positive on 50-g proceed to the next step using 75 or 100-g. Hence, the overall prevalence of GDM based on one-step screening method will be higher. This is consistent with the literature where the two-step screening method is less sensitive than the one-step screening method in diagnosing GDM, and the two-step screening method will miss approximately 25% of cases [162]. In view of one-step screening method is more practical, cost effective and more convenient [161, 163]. Hence, it is a more advantage to use one-step method instead of two-steps method in diagnosing GDM. Having say so till now there is no consensus for use of the one-step versus two-step screening method among national and international organizations. Recent Cochrane review in 2017 reported that there is insufficient evidence to suggest which strategy is best for diagnosing GDM [164].

The majority of the included studies in this review were conducted in hospitals (12.0%). 71 studies had conducted the screening for GDM during antenatal visits at the hospitals. Meanwhile, 13 studies were conducted in the community hospitals, which mostly involved the authorities in healthcare such as the MOH to perform wide coverage screening for GDM at national, state or regional level.

Taiwan had the highest prevalence of GDM (38.6%). The study conducted in Taiwan had a small sample size (*n* = 132) and the pregnant women were older (mean age of 32) and the chosen study location was mainly inhabited by aboriginal tribes. On top of that the data were collected using 2 different diagnostic criteria. The 100 g three-hour OGTT test was used before 2012 and 75 g OGTT test with a better sensitivity was used since 2012. As we know the prevalence of GDM may be varied according to different diagnostic criteria used [165]. Hong Kong also had a high prevalence of GDM (32.5%) due to the screening was performed at referral hospital for GDM cases, and these GDM group are those in advance age as the mean age of the study population was 34 and higher parity. The prevalence of GDM in Taiwan and Hong Kong were derived from only one study each and hence the reported prevalence are not representable for the true burden of GDM in their countries.

The risk factors of GDM was analysed in this current review. Those with multiparity ≥2, previous history of GDM, congenital anomalies, stillbirth, abortion, preterm delivery, macrosomia, having concurrent PIH, PCOS, age ≥ 25, BMI ≥25, and family history of diabetes are the significant risk factors predictive of GDM in current pregnancy (OR values ranged from 1.90 to 8.42). Most of the guidelines, including those of ADA in 2016, recommend universal screening for GDM in second trimester [166]. Other organizations, such as NICE in 2015, recommend screening for GDM using risk factors at the booking appointment. The risk factors considered by NICE in 2015 are BMI ≥ 30, a history of macrosomia of 4.5 kg or more, previous gestational diabetes, a family history of diabetes, or belonging to an ethnic minority with a high prevalence of gestational diabetes such as South Asian and Middle Eastern [167]. In Malaysia, pregnant women age ≥ 25 together with risk factors should be screened for GDM at booking. The risk factors for GDM are those with BMI ≥ 27, previous history of GDM, macrosomia (birth weight > 4 kg), bad obstetric history, glycosuria ≥2 + on two occasions, first degree relative with diabetes mellitus, concomitant obstetrics problems such as hypertension or pregnancy-induced hypertension, polyhydramnios and current use of corticosteroids [168]. While in France, the identified risk factors requiring the search for GDM are maternal age ≥ 35 years, BMI ≥ 25, history of diabetes in first-degree relatives, personal history of GDM or GDM [169].

Our study showed that those with history of previous GDM have 3.5 times odds more likely to develop GDM compare those without history of previous GDM. This finding is consistent with previous study [28, 114].

History of congenital anomalies have 4.3 times odds more likely to develop GDM compare those without history of congenital anomalies. This finding is consistent with previous study [28, 93]. Similarly, to those with history of macrosomia and PIH have 4 times and 3 times for odds to have higher insulin resistance. This is consistent with the previous finding [84, 91].

Polycystic ovarian syndrome (PCOS) is a common cause of insulin resistance [104, 151]. Women with PCOS have higher risk of developing GDM [104, 151] and this is consistent with our study (OR 2.33, 95% CI 1.72–3.17).

BMI is commonly used in risk-based screening for GDM. Prevalence of GDM is also increased with increasing pre-pregnancy BMI [170]. For instance, prevalence of GDM was highest among Asian women with BMI ≥ 30 kg/m^2^ (13.78%), followed by BMI ≥ 25 kg/m^2^ (10.22%) and BMI ≥ 20 kg/m^2^ (6.09%). In this current review, we used a BMI cut-off of ≥25 kg/m^2^ and found the odds ratio for GDM is 3.39 (95% CI2.92–3.93). Our result is consistent with previous studies where the odds of BMI ≥25 kg/m^2^ for GDM ranged from 2.78 (95% CI: 2.60–2.96) to 3.56 (95% CI: 3.05–4.21) [65, 171].

A BMI ≥ 25 kg/m^2^ has a lower sensitivity (24.9%) but a good specificity (88.7%) in comparison to using a cut-off level of BMI ≥ 21 kg/m^2^ which has a higher sensitivity of 68.4% but a lower specificity of 53.6% [170]. Literature suggests a BMI ≥25 kg/m^2^ is more suitable to be used among African-American women as the sensitivity (46.2%) and specificity (81.5%) are higher. A BMI ≥21.0 kg/m^2^ would be recommended as cut off threshold to screen GDM with a better sensitivity however BMI *I* ≥ 25.0 kg/m^2^ was the most commonly used threshold among the included studies [170].

Obesity is one of the main factors in the development of diabetes and GDM [64, 172]. BMI is a commonly used method to measure the severity of obesity [173]. However, the cut-off point used to diagnose obesity is different between western and Asian countries [170]. For example, prevalence of GDM was highest among Asian women with BMI ≥ 30 kg/m^2^ (13.78%), followed by BMI ≥ 25 kg/m^2^ (10.22%) and BMI ≥ 20 kg/m^2^ (6.09%). In this current review, we have employed a BMI cut-off of ≥25 kg/m^2^ and found the odds ratio for GDM is 3.27 (95% CI2.81–3.80). Our results are consistent with previous studies in which the odds of BMI ≥25 kg/m^2^ for GDM ranged from 2.78 (95% CI: 2.60–2.96) to 3.56 (95% CI: 3.05–4.21) [65, 171].

Maternal age is an established risk factor for GDM, but there is no consensus on age’s relation to increased risk of GDM [174]. ADA recommended the lowest cutoff of ≥25 years to screen for GDM as early as possible [43]. This is supported by our results showing that the odds of GDM by age ≥ 25 is OR 2.17 (95% CI 1.96–2.41), and consistent with previous study findings showing that screening for GDM among patients aged 25 years and above with other risk factors indeed has a higher predictive value in identifying GDM [175].

According to previous studies, family history of diabetes (particularly in a first-degree relative) increases the risk for GDM [64, 66]. Onset of GDM has a familial tendency and this potentially suggests that there is a genetically predisposition to develop GDM [176–178]. In current review, family history of diabetes has OR 2.77(95% CI 2.22–3.47) of GDM. Our results are consistent with a previous study in which the odds of family history of diabetes for GDM among Iranian women was determined to be OR 3.46 (95%CI 2.8–4.27) [179].

The strength of this review paper is that it not only included more countries, including India and countries in Middle East which were both not included in previous reports. Furthermore, the articles with poor quality in STROBE were excluded to maintain the reliability of findings of current review.

Our meta-analysis has the following limitations. Firstly, we are aware that the studies included in this meta-analysis are not a true reflection of the Asian population. Although there were 24 studies in the meta-analysis come from India, they only contributed 17,049 patients out of the general population of 1.3 billion in India. Similarly, the 8 Chinese studies only contributed 156,942 patients out of 1.4 billion in China. Based on the inclusion criteria, we have recruited the above 32 studies in this review. Thus, we must interpret the results of this meta-analysis cautiously within the context of their limitations. Secondly, there was a high heterogeneity in our result. This could be due to different diagnostic criteria and screening methods used by different countries. This high heterogeneity may also be due to the different population characteristics as 20 countries were included in this meta-analysis. Thirdly, this meta-analysis included manuscripts from the inception to 2018, covering a vast range of clinical and diagnostic criteria and practice changes. The threshold value of two-hour in one-step 75-g method and three-hour in 100-g two-steps methods are reduced over time, increasing the identification rates of GDM cases over time. Therefore, changes of threshold value to identify GDM could inevitably cause high heterogeneity to the results. Finally, studies with small sample size were also included in this meta-analysis. Hence the result of this meta-analysis may suffer from high variability. Therefore, some estimates of the meta-analysis could be influenced by heterogeneity between the studies.

## Conclusions

Our current study provides an estimation of the prevalence and risk factors of GDM in Asia. Our study shows that the pooled estimation of prevalence was 11.5%. We have identified the following risk factors of developing GDM: multiparity≥2; previous history of GDM; congenital anomalies; stillbirth; abortion; preterm delivery; macrosomia; concurrent PIH; PCOS; age ≥ 25; BMI ≥25; and family history of diabetes.

It is important that the risk factors for GDM are recognized in order the clinicians are able to identify those at risk of getting GDM for early diagnosis and further intervention. We recommend that clinicians screen for GDM as early as possible among those with risk factors using one-step screening method instead of two-step screening method. If the results are negative, the test should be repeated in between 24 and 28 weeks of gestation.

## Appendix 1

**Table 3:** Screening criteria for the diagnosis of Gestational Diabetes Mellitus

Diagnostics criteria	Steps	OGTT	No. abnormal	Fasting mg/dl (mmol/l)	1 H mg/dl (mmol/l)	2 H mg/dl (mmol/l)	3H mg/dl (mmol/l)
O′ Sullivan 1964 [49]	2	100 g	≥ 2	90 (5)	165 (9.2)	145 (8.1)	125 (6.9)
NDDG 1979 [50]	2	100 g	≥ 2	105 (5.8)	190 (10.6)	165 (9.2)	145 (8.0)
CC 1982 [51]	2	100 g	≥ 2	95 (5.3)	180 (10)	155 (8.6)	140 (7.8)
EASD 2012 [52]	1	75 g	≥ 1	108 (6)	–	162 (9)	–
ACOG [53]	2	100 g	≥ 2	95 (5.30	180 (10)	155 (8.6)	140 (7.8)
ADIPS 1998 [54]	1	75 g	≥ 1	100 (5.5)	–	144 (8.0)	–
IADPSG 2010 [55]	1	75 g	≥ 2	92 (5.1)	180 (10)	153 (8.5)	–
DIPSI [25]	1	75 g	≥ 1	–	–	140 (7.8)	–
JDS [56]	1	75 g	≥ 2	126 (7)	–	200 (11.1)	–
China MOH [57]	1	75 g	≥ 1	92 (5.1)	180 (10)	153 (8.5)	–
ICD-10 O24.4 (58)	2	75 g	≥ 1	92 (5.1)	180 (10)	153 (8.5)	–
ADA 1997 [59]	1	75 g	≥ 1	126 (7)	–	200 (11.1)	–
ADA 2002 [37]	1	75 g	≥ 1	126 (7)	–	200 (11.1)	
ADA 2003 [38]	2	100 g	≥ 2	95 (5.3)	180 (10)	155 (8.6)	140 (7.8)
ADA 2004 [39]	2	100 g	≥ 1	95 (5.3)	180 (10)	155 (8.6)	140 (7.8)
ADA 2011 [40]	1	75 g	≥ 1	92 (5.1)	–	–	–
ADA 2012 [41]	1	75 g	≥ 1	92 (5.1)	180 (10)	153 (8.5)	–
ADA 2014 [42]	1	75 g	≥ 1	–	180 (10)	153 (8.5)	–
WHO 1980 [43]	1	75 g	≥ 1	140 (7.8)	–	200 (11.1)	–
WHO 1998 [44]	1	75 g	≥ 1	126 (7)	–	200 (10)	–
WHO 1985 [45]	1	75 g	≥ 1	–	–	140 (7.8)	–
WHO 1999 [44]	1	75 g	≥ 1	126 (7)	–	140 (7.8)	–
WHO 2006 [46]	1	75 g	≥ 1	126 (7)	180 (10)	200 (11.1)	–
WHO 2013 [47]	1	75 g	≥ 1	92 (5.1)	180 (10)	153 (8.5)	–
CDA 2008 [48]	1	75 g	≥ 2	95 (5.3)	190 (10.6)	160 (8.9)	–

*ACOG* The American College of Obstetricians and Gynecologists, *ADA* American Diabetes Association, *ADIPS* Australian Diabetes in Pregnancy Society, *CC* Carpenter-Coustan, *CDA* Canadian Diabetes Association, *DIPSI* Diabetes in Pregnancy Study group of India, *EASD* European Association for the Study of Diabetes, *IADPSG* International Association of the Diabetes and Pregnancy Study Groups, *ICD* International Classification of Diseases, *JDS* Japan Diabetes Society, *NDDG* National Diabetes Data Group, *OGTT* Oral Glucose tolerance Test, *WHO* World Health Organization, *MOH* Ministry of Health

## Appendix 2

**Table 4:** Search terms used for final search 22 August 2017

Searches	Search terms	Pubmed	Ovid	Sciencedirect	Scopus
# 1	Incidence	2,424,449	230,603	194,404	2,761,766
# 2	Prevalence	2,583,495	253,174	141,405	2,490,683
# 3	Risk factor	1,264,673	726,298	209,966	4,281,945
# 4	Diabetes in pregnancy	36,326	10,331	4542	135,425
# 5	Gestational diabetes mellitus	18,375	8665	2156	46,028
# 6	Asia	755,317	26,037	52,858	1,413,577
# 7	#1 OR #2	2,812,427	461,976	322,237	4,476,331
# 8	#4 OR #5	36,326	18,121	5314	145,571
#9	#7 AND #8 AND #3 AND #6	608	630	318	838

## Appendix 3

**Table 5:** Assessment of risk of bias of included studies by STROBE Checklist

	1a	1b	2	3	4	5	6a	6b	7	8	9	10	11	12a	12b	12c	12d	12e	13a	13b	13c	14a	14b	14c	15	16a	16b	16c	17	18	19	20	21	22
Alfadhli et al., 2015	1	1	1	1	1	1	1	1	1	1	0	1	1	1	1	0	1	0	1	1	1	1	0	1	1	1	1	0	0	1	1	1	1	0
Ali et al., 2016	1	1	1	1	1	1	1	1	1	1	0	0	1	1	1	0	1	0	1	0	0	1	1	1	1	1	1	0	0	1	1	1	1	1
Al-Kuwari et al., 2011	1	1	1	1	1	1	1	0	1	1	0	0	1	1	1	0	1	0	1	1	0	1	0	1	1	1	1	0	0	1	0	1	1	0
Al-Rowaily et al., 2010	0	1	1	1	0	1	1	0	1	1	0	1	1	1	1	0	1	0	1	0	0	1	0	0	1	1	1	0	0	1	1	1	1	0
Al-Rubeaan e al., 2014	1	1	1	1	1	1	1	1	1	1	0	1	1	1	0	0	1	0	1	1	1	1	0	1	1	1	1	0	0	1	1	1	1	1
Al-Shawaf et al., 1988	0	1	1	1	0	1	1	1	1	1	0	0	1	1	0	0	1	0	1	0	0	1	0	1	1	0	1	0	0	1	0	1	1	0
Aziz et al., 2017	1	1	1	1	1	1	1	1	1	1	0	1	0	0	0	0	0	0	1	0	0	1	0	1	1	0	1	0	0	1	0	1	1	0
Bener et al., 2011	1	1	1	1	1	1	1	1	1	1	0	1	1	1	1	0	1	0	1	0	0	1	0	1	1	1	1	0	0	1	0	1	1	0
Bhatt et al., 2015	0	1	1	1	0	1	0	1	1	1	0	1	1	1	0	0	0	1	1	0	0	1	0	1	1	1	1	0	0	1	1	1	1	1
Cheuk et al., 2016	1	1	1	1	1	1	1	0	1	1	0	0	1	1	1	0	1	0	1	1	1	1	0	1	1	0	0	0	0	1	1	1	1	0
Chodick et al., 2010	1	1	1	1	1	1	1	0	1	1	0	0	1	1	1	0	1	0	1	1	0	1	1	1	1	1	1	1	0	1	1	1	1	0
Dahiya et al., 2014	0	1	1	1	1	1	1	1	1	1	0	0	1	1	0	0	1	0	1	0	0	1	0	1	1	1	1	0	0	1	0	1	1	0
Das et al., 2004	1	1	0	1	1	1	1	1	1	1	0	1	0	0	0	0	1	0	1	1	0	1	0	1	1	0	0	0	0	1	0	1	1	0
De Seymour et al., 2016	1	1	1	1	1	1	1	1	1	1	0	1	1	1	1	1	0	0	1	1	1	1	1	1	1	1	1	0	0	1	1	1	1	1
Deerochanawong et al., 1996	0	1	1	1	0	1	1	1	1	1	0	0	1	1	0	0	0	0	1	0	0	1	0	1	1	1	1	1	0	1	0	1	1	0
Garshasbi et al., 2008	1	1	1	1	1	1	1	1	1	1	0	1	1	1	1	0	1	0	1	1	1	1	0	1	1	1	1	0	0	1	0	1	1	0
Gracelyn and Saranya2016	1	1	1	1	1	1	1	1	1	1	1	0	1	1	0	0	1	0	1	0	0	1	0	1	1	1	1	0	0	1	0	1	1	1
Haideagh et al., 2005	0	1	1	1	0	1	1	0	1	1	0	1	1	0	0	0	1	0	1	1	0	1	1	1	1	0	1	0	0	1	0	1	1	0
Hariharan et al., 2017	1	1	1	1	1	1	1	1	1	1	0	1	0	1	0	0	1	0	1	0	0	0	0	1	1	0	0	0	0	1	1	1	1	0
Herath et al., 2016	1	1	1	1	1	1	1	1	1	1	0	0	1	1	0	0	1	1	1	0	0	1	1	1	1	0	1	0	1	1	0	1	1	0
Hirst et al., 2012	1	1	1	1	1	1	1	1	1	1	1	0	1	1	1	1	0	1	1	1	1	1	1	1	1	1	1	0	1	0	1	1	1	1
Hossain et al., 2017	1	1	1	1	1	1	1	0	1	1	0	0	1	1	1	0	1	1	1	0	0	1	0	1	1	1	1	0	1	1	1	1	1	0
Hossein-Nezhad et al.,2007	1	1	1	1	1	1	1	1	1	1	0	1	1	1	1	0	1	0	1	0	0	1	0	1	1	1	1	0	0	1	0	1	1	0
Iqbal et al., 2007	1	1	1	1	1	1	1	0	1	1	0	0	1	1	1	0	1	0	1	1	0	1	1	1	1	1	1	0	0	1	1	1	1	1
Jadhav and Wankhede 2017	0	1	1	1	0	1	1	1	1	1	1	0	0	0	0	0	0	0	1	0	0	1	0	1	1	0	1	0	0	1	0	1	1	1
Jang et al., 1998	1	1	1	1	1	1	1	1	1	1	0	1	1	1	1	0	1	0	1	1	0	1	0	1	1	1	1	0	0	1	0	1	1	1
Jesmin et al., 2014	1	1	1	1	1	1	0	1	1	1	1	0	1	1	1	0	0	0	0	0	0	0	1	0	0	1	1	0	0	1	1	1	1	1
Kalra et al., 2013	0	1	1	1	0	1	1	1	1	1	0	1	1	0	0	0	0	1	1	0	0	1	0	1	1	0	1	0	0	1	0	1	1	0
Kalyani et al., 2014	0	1	1	1	0	1	1	1	1	1	1	0	0	0	0	0	0	0	1	0	0	1	0	1	1	0	1	0	0	1	0	1	1	1
Khwaja et al., 1989	0	0	1	1	0	1	1	1	1	1	0	1	1	1	0	0	0	0	1	0	0	1	1	1	1	0	1	0	0	1	0	1	1	0
Koo et al., 2016	1	1	1	1	0	1	1	0	1	0	0	0	0	1	1	0	1	0	1	0	0	1	0	1	1	1	1	0	0	1	1	1	1	1
Krishnaveni et al., 2007	0	1	1	1	0	1	1	1	1	1	0	1	1	0	0	0	1	0	1	0	0	1	0	1	1	1	1	0	0	1	0	1	1	1
Leng et al., 2016	1	1	1	1	1	1	1	1	1	1	0	1	1	1	1	0	1	0	1	0	0	1	0	1	1	1	1	0	1	1	1	1	1	1
Li et al., 2014	1	1	1	1	1	1	1	1	1	1	0	1	1	1	1	0	1	0	1	0	0	1	0	1	1	1	1	0	0	1	1	1	1	1
Li et al., 2016	1	1	1	1	1	1	1	0	1	1	0	0	1	1	1	0	1	1	1	0	0	1	0	1	1	1	1	0	1	1	1	1	1	0
Lin et al., 2015	1	1	1	1	0	1	1	0	1	1	0	0	0	0	0	0	1	0	1	1	0	1	0	1	1	1	1	0	0	1	1	1	1	0
Maegawa et al., 2003	0	1	1	1	0	1	0	1	1	1	0	1	1	1	1	0	0	1	1	0	0	1	1	1	1	0	1	0	1	1	1	1	1	0
Makwana et al., 2017	0	1	1	1	0	1	1	0	1	1	0	0	1	0	0	0	0	0	1	0	0	1	0	1	1	0	1	0	0	1	0	1	1	1
Mizuno et al., 2016	1	1	1	1	0	1	1	1	1	1	0	1	1	1	0	0	1	0	1	1	1	1	0	1	1	1	1	0	0	1	1	1	1	1
Mohammadzadeh et al., 2015	0	1	1	1	1	1	1	1	1	1	0	0	1	1	1	0	1	0	1	1	0	1	1	1	1	1	1	0	0	1	1	1	1	1
Moradi et al., 2015	0	1	1	1	0	1	1	1	1	1	0	0	1	1	0	0	1	0	1	0	0	1	0	1	1	0	1	0	0	1	1	1	1	0
Mustafa 2015	1	1	1	1	1	1	1	1	1	1	0	0	1	0	0	0	0	0	0	0	0	1	0	1	1	0	1	0	0	1	1	1	1	0
Nayak et al., 2013	1	1	1	1	1	1	1	1	1	1	0	1	1	1	0	0	1	0	1	0	0	1	0	1	1	0	0	0	0	1	1	1	1	1
Nielsen et al., 2016	1	1	1	1	1	1	1	0	1	1	0	1	1	1	1	0	0	1	1	0	0	1	0	1	1	1	1	0	1	1	1	1	1	1
Parhofer et al., 2013	1	1	1	1	0	1	1	0	1	1	0	0	1	1	0	0	0	0	1	1	1	1	0	1	1	0	0	0	0	1	1	1	1	1
Pirjani et al.,2016	1	1	1	1	1	1	1	0	0	1	0	0	1	1	1	0	1	0	0	0	0	1	0	1	1	1	1	0	1	1	1	1	1	0
Raja et al., 2014	1	1	1	1	1	1	1	0	1	1	0	1	1	1	0	0	0	0	0	0	0	1	0	1	1	0	0	0	0	1	0	1	1	0
Rajput et al., 2013	1	1	1	1	0	1	1	0	1	1	0	1	0	1	0	0	1	0	1	0	0	1	0	1	1	1	1	0	0	1	0	1	1	0
Rajput et al., 2014	0	1	1	1	1	1	1	1	1	1	0	1	1	1	0	0	0	0	0	0	0	1	0	1	1	1	1	0	0	1	0	1	1	0
Rao et al., 2015	0	1	1	1	0	1	1	1	1	1	0	0	1	0	0	0	0	0	1	0	0	1	0	1	0	0	1	0	0	1	0	1	1	0
Saisho et al., 2013	0	1	1	1	1	1	1	1	1	1	0	1	1	0	0	0	0	0	0	0	0	1	0	1	1	0	1	0	0	1	1	1	1	0
Sayeed et al., 2005	0	1	1	1	1	1	1	1	1	1	0	1	1	1	0	1	1	0	1	1	1	1	1	1	1	1	1	0	0	1	1	1	1	1
Sella et al., 2013	1	1	1	1	1	1	1	0	1	1	0	1	1	1	1	0	0	0	1	1	0	1	0	1	1	1	1	0	0	1	0	1	1	0
Sella et al., 2011	1	1	1	1	1	1	1	0	1	1	0	0	0	1	1	1	1	0	1	0	0	1	0	1	1	1	1	0	0	1	1	1	1	0
Shahbazian et al.,2016	1	1	1	1	1	1	1	1	1	1	0	0	0	1	1	0	1	0	1	0	0	1	0	1	1	1	1	0	0	1	1	1	1	1
Shaman et al., 2015	1	1	1	1	1	1	1	1	1	1	0	1	1	1	0	0	1	0	1	0	0	1	0	1	1	0	0	0	0	1	0	1	1	1
Shamsuddin et al., 2001	1	1	0	1	1	1	1	0	1	1	0	0	0	1	0	0	1	1	1	1	0	1	0	1	1	0	0	0	1	1	0	1	1	0
Shang et al., 2014	0	1	1	1	0	1	1	0	1	1	0	0	1	1	1	0	1	0	1	1	1	1	0	0	1	1	1	0	0	1	0	1	1	1
Sharma et al., 2016	1	1	1	1	1	1	1	0	1	1	0	0	1	1	0	0	1	1	1	0	0	1	0	1	1	0	1	0	1	1	1	1	1	1
Shimodaira et al., 2016	1	1	1	1	1	1	1	1	1	1	1	1	1	1	1	1	0	0	1	1	0	1	0	1	1	1	1	0	0	1	1	1	1	0
Shrestha and Chawla 2011	1	1	1	1	1	1	0	1	1	1	0	1	1	1	0	0	0	0	1	0	0	1	0	1	1	0	1	0	0	1	0	1	1	0
Shridevi et al., 2015	0	1	1	1	0	1	1	1	1	1	0	1	1	1	0	0	1	0	1	0	0	1	0	1	1	0	1	0	0	1	0	1	1	1
Singh and Uma 2013	1	1	1	1	1	1	1	0	1	1	0	0	1	1	1	0	1	0	1	0	0	1	0	1	1	0	0	0	0	1	0	1	1	0
Siribaddana et al., 1998	1	1	1	1	1	1	1	1	1	1	0	0	1	0	0	0	1	0	1	0	0	1	0	1	1	0	1	0	0	1	0	1	1	0
Soheilykhak et al., 2010	1	1	1	1	1	1	1	0	1	1	1	0	1	1	0	0	1	0	1	1	0	1	0	1	1	1	1	1	0	1	0	1	1	1
Song et al., 2017	1	1	1	1	1	1	1	1	1	1	0	1	1	1	1	1	0	0	1	0	0	1	1	1	1	1	1	0	0	1	1	1	1	1
Srichumchit et al., 2015	1	1	0	1	1	1	1	1	1	1	0	0	0	1	0	0	1	0	1	1	1	1	0	1	1	1	1	0	0	1	1	1	1	1
Sudasinghe et al., 2016	1	1	1	1	1	1	1	1	0	1	0	1	0	1	0	0	1	0	1	1	0	1	0	1	1	0	0	0	0	1	1	1	1	1
Suntorn and Panichkul 2015	1	1	1	1	1	1	1	0	1	1	0	0	1	1	1	0	1	0	1	1	0	1	0	1	1	0	1	0	0	1	1	1	1	0
Swaroop et al., 2015	1	1	1	1	1	1	1	0	1	1	0	0	0	1	0	0	0	0	1	0	0	1	0	1	1	0	0	0	0	1	0	1	1	1
Tan et al., 2009	1	1	1	1	1	1	1	1	1	1	0	1	1	1	1	0	1	0	1	1	1	1	0	1	1	1	1	0	0	1	1	1	1	0
Thapa et al., 2015	0	1	1	1	1	1	1	1	1	1	0	1	1	1	0	0	0	0	1	0	0	1	0	1	1	0	1	0	0	1	1	1	1	1
Thathagari et al., 2016	1	1	1	1	1	1	1	1	1	1	0	1	1	1	1	0	1	0	1	0	0	1	0	1	1	1	1	0	0	1	0	1	1	1
Tran et al., 2013	1	1	1	1	1	1	1	1	1	1	0	1	1	1	1	0	1	1	1	1	0	1	1	0	1	0	0	0	1	1	1	1	1	1
Tripathi et al., 2011	1	1	1	1	1	1	1	1	1	1	0	1	1	0	0	0	1	0	1	0	1	1	0	1	1	1	1	0	0	1	0	1	1	0
Vakili et al., 2016	1	1	1	1	1	1	1	1	1	1	0	1	1	1	0	0	1	0	1	0	0	1	0	1	1	1	0	0	0	1	1	1	1	0
Wagaarachchi et al., 2001	1	1	1	1	0	1	1	1	1	0	0	1	0	1	0	0	1	0	1	1	0	1	0	1	1	0	0	0	0	1	0	1	1	1
Wahabi et al., 2017	0	1	1	1	1	1	1	1	1	1	0	0	1	1	0	0	1	0	1	1	0	1	0	1	1	1	1	0	0	1	1	1	1	1
Warunpitikul and Aswakul 2014	1	1	1	1	1	1	1	1	1	1	0	0	1	1	0	0	1	0	1	0	0	1	0	1	1	1	1	0	0	1	0	1	1	1
Yang et al., 2013	1	1	0	1	0	1	1	1	1	1	0	1	1	1	1	0	1	0	1	0	0	1	0	1	1	0	0	0	0	1	1	1	1	1
Zargar et al., 2004	1	1	1	1	1	1	1	0	1	1	0	0	1	1	0	0	1	0	1	0	0	1	0	1	1	1	1	0	0	1	0	1	1	0
Zhang et al., 2011	1	1	1	1	1	1	1	1	0	1	0	1	1	1	0	0	1	0	1	0	0	1	0	1	1	1	1	0	0	1	1	1	1	1
Zhang et al., 2015	1	1	1	1	1	1	1	1	1	1	0	1	1	1	1	0	1	1	1	0	0	1	0	1	1	1	1	0	1	1	1	1	1	1
Zhu et al., 2017	0	1	1	1	0	1	1	1	1	1	0	1	1	1	0	0	1	0	1	0	0	1	0	1	1	1	1	0	0	1	1	1	1	1

1(Presence of item)

0(Absence of item)

^†^Quality is defined by a STROBE score ≥ 14/22 (good) and < 14/22 (poor)

## Appendix 4

**Table 6:** Characteristics of Included studies

Author, year	Country	Association	Diagnostic criteria	Study Setting	Screening Methods	Screening dosage	GDM +	Sample size	Prevalence
Alfadhli et al., 2015	Saudi Arabia	IADPSG	IADPSG	Hospital	1	75 g	292	573	51.0
Ali et al., 2016	Yemen	ADA	ADA 2002	Hospital	1	75 g	16	311	5.1
Al-Kuwari et al., 2011	Qatar	ADA	ADA 2004	Hospital	2	50 g, 75 g	61	597	10.2
Al-Rowaily et al., 2010	Saudi Arabia	WHO	WHO 1999	Hospital	1	75 g	79	633	12.5
Al-Rubeaan e al., 2014	Saudi Arabia	ADA	ADA 2011	Community	1	75 g	201	529	38.0
Al-Shawaf et al., 1988	Saudi Arabia	WHO	WHO 1985	Hospital	2	75 g	41	1102	3.7
Aziz et al., 2017	India	CC	CC	Hospital	1	100 g	56	700	8.0
Bener et al., 2011	Qatar	WHO	WHO 2006	Hospital	1	75 g	262	1608	16.3
Bhatt et al., 2015	India	DIPSI	DIPSI	Community	1	75 g	94	989	9.5
Cheuk et al., 2016	Hong Kong	WHO	WHO 1999	Hospital	1	75 g	169	520	32.5
Chodick et al., 2010	Israel	CC	CC	Community	1	100 g	11,270	185,416	6.1
Dahiya et al., 2014	India	DIPSI	DIPSI	Hospital	1	75 g	35	500	7.0
Das et al., 2004	India	NDDG	NDDG	Hospital	2	50 g, 100 g	12	300	4.0
De Seymour et al., 2016	Singapore	WHO	WHO 1999	Hospital	1		160	909	17.6
Deerochanawong et al., 1996	Thailand	WHO	WHO 1985	Hospital	2	50 g, 75 g	111	709	15.7
Garshasbi et al., 2008	Iran	CC	CC	Hospital	2	50 g, 100 g	124	1804	6.9
Gracelyn and Saranya2016	India	ADA	ADA 2014	Hospital	1	75 g	59	500	11.8
Haideagh et al., 2005	Iran	CC	CC	Community	2	50 g, 100 g	62	800	7.8
Hariharan et al., 2017	India	WHO	WHO 2013	Hospital	1	75 g	16	135	11.9
Herath et al., 2016	Sri lanka	IADPSG	IADPSG	Hospital	1	75 g	105	452	23.2
Hirst et al., 2012	Vietnam	IADPSG	IADPSG	Hospital	1	75 g	550	2702	20.4
Hossain et al., 2017	Pakistan	DIPSI	DIPSI	Hospital	1	75 g	78	1030	7.6
Hossein-Nezhad et al.,2007	Iran	CC	CC	Hospital	2	50 g, 100 g	114	2416	4.7
Iqbal et al., 2007	Pakistan	ADA	ADA 2004	Hospital	2	75 g, 100 g	49	612	8.0
Jadhav and Wankhede 2017	India	DIPSI	DIPSI	Hospital	1	75 g	80	1000	8.0
Jang et al., 1998	South Korea	NDDG	NDDG	Hospital	2	50 g, 100 g	173	8863	2.0
Jesmin et al., 2014	Bangladesh	WHO	WHO 1999	Hospital	2	50 g, 75 g	112	1149	9.7
Kalra et al., 2013	India	DIPSI	DIPSI	Hospital	1	75 g	33	500	6.6
Kalyani et al., 2014	India	WHO	WHO 1999	Hospital	1	75 g	25	300	8.3
Khwaja et al., 1989	Saudi Arabia	WHO	WHO 1985	Hospital	1	50 g	50	455	11.0
Koo et al., 2016	South Korea	ICD-10	ICD-10	Community			98,403	1,306,281	7.5
Krishnaveni et al., 2007	India	CC	CC	Hospital	1	100 g	21	524	4.0
Leng et al., 2016	China	IADPSG	IADPSG	Community	2	50 g, 75 g	840	11,450	7.3
Li et al., 2014	China	ADA	ADA 2012	Hospital	1	75 g	69	539	12.8
Li et al., 2016	China	IADPSG	IADPSG	Hospital	1	75 g	48	327	14.7
Lin et al., 2015	Taiwan	ADA	ADA undefined	Hospital	1	75 g or 100 g	51	132	38.6
Maegawa et al., 2003	Japan	JDS	JDS	Hospital	2	50 g or 75 g	22	749	2.9
Makwana et al., 2017	India	DIPSI	DIPSI	Hospital	1	75 g	38	476	8.0
Mizuno et al., 2016	Japan	JDS	JDS	Hospital	1	75 g	204	8874	2.3
Mohammadzadeh et al., 2015	Iran	CC	CC	Hospital	2	50 g, 100 g	62	1276	4.9
Moradi et al., 2015	Iran	WHO	WHO 2006	Hospital	1	75 g	44	290	15.2
Mustafa 2015	Bangladesh	WHO	WHO 1999	Hospital	1	75 g	102	1489	6.9
Nayak et al., 2013	India	IADPSG	IADPSG	Hospital	1	75 g	83	304	27.3
Nielsen et al., 2016	India	DIPSI	DIPSI	Hospital	1	75 g	659	4053	16.3
Parhofer et al., 2013	Turkmenistan	ADA	ADA undefined	Hospital	2	50 g, 75 g	109	1620	6.7
Pirjani et al.,2016	Iran	ADA	ADA 2012	Hospital	1	75 g	78	256	30.5
Raja et al., 2014	India	DIPSI	DIPSI	Community	1	75 g	24	306	7.8
Rajput et al., 2013	India	ADA	ADA 2004	Hospital	1	75 g	43	607	7.1
Rajput et al., 2014	India	WHO	WHO 1999	Community	1	75 g	127	913	13.9
Rao et al., 2015	India	DIPSI	DIPSI	Hospital	1	75 g	5	200	2.5
Saisho et al., 2013	Japan	JDS	JDS	Hospital	2	50 g, 75 g	15	62	24.2
Sayeed et al., 2005	Bangladesh	WHO	WHO 1999	Community	1	75 g	12	147	8.2
Sella et al., 2011	Israel	CC	CC	Hospital	2	50 g, 100 g	11,264	185,315	6.1
Sella et al., 2013	Israel	ADA	ADA 2003	Community	1	100 g	14,288	367,247	3.9
Shahbazian et al.,2016	Iran	IADPSG	IADPSG	Hospital	1	75 g	224	750	29.9
Shaman et al., 2015	Saudi Arabia	IADPSG	IADPSG	Hospital	1	75 g	175	850	20.6
Shamsuddin et al., 2001	Malaysia	WHO	WHO 1985	Hospital	1	75 g	191	768	24.9
Shang et al., 2014	China	IADPSG	IADPSG	Hospital	1	75 g	612	3083	19.9
Sharma et al., 2016	India	IADPSG	IADPSG	Hospital	1	75 g	74	417	17.7
Shimodaira et al., 2016	Japan	ADA	ADA 2004	Hospital	2	50 g, 75 g	149	5424	2.7
Shrestha and Chawla 2011	Nepal	CC	CC	Hospital	2	50 g, 100 g	12	1598	0.8
Shridevi et al., 2015	India	DIPSI	DIPSI	Hospital	2	50 g, 75 g	23	200	11.5
Singh and Uma 2013	India	DIPSI	DIPSI	Hospital	1	75 g	23	400	5.8
Siribaddana et al., 1998	Sri lanka	WHO	WHO 1985	Hospital	2	50 g, 75 g	40	721	5.5
Soheilykhak et al., 2010	Iran	CC	CC	Hospital	2	50 g, 100 g	110	1071	10.3
Song et al., 2017	China	IADPSG	IADPSG	Hospital	1	75 g	1005	6886	14.6
Srichumchit et al., 2015	Thailand	NDDG	NDDG	Hospital	2	50 g, 100 g	1350	21,771	6.2
Sudasinghe et al., 2016	Sri lanka	WHO	WHO 1999	Community	1	75 g	194	1400	13.9
Suntorn and Panichkul 2015	Thailand	IADPSG	IADPSG	Hospital	1	75 g	71	325	21.8
Swaroop et al., 2015	India	DIPSI	DIPSI	Hospital	1	75 g	22	225	9.8
Tan et al., 2009	Malaysia	WHO	WHO 1999	Hospital	2	50 g, 75 g	168	1368	12.3
Thapa et al., 2015	Nepal	WHO	WHO 1999	Hospital	1	75 g	14	564	2.5
Thathagari et al., 2016	India	NDDG	NDDG	Hospital	2	50 g, 100 g	42	800	5.3
Tran et al., 2013	Vietnam	WHO	WHO 1999	Hospital	1	75 g	674	2772	24.3
Tripathi et al., 2011	India	CC	CC	Hospital	2	50 g, 100 g	10	700	1.4
Vakili et al., 2016	Iran	ADA	ADA 2004	Community	1	75 g	328	1209	27.1
Wagaarachchi et al., 2001	Sri lanka	WHO	WHO 1980	Hospital	1	75 g	41	1004	4.1
Wahabi et al., 2017	Saudi Arabia	WHO	WHO 2013	Hospital	1	75 g	2354	9723	24.2
Warunpitikul and Aswakul 2014	Thailand	CC	CC	Hospital	2	50 g, 100 g	340	1363	24.9
Yang et al., 2013	South Korea	CC	CC	Hospital	2	50 g, 100 g	269	1163	23.1
Zargar et al., 2004	India	CC and WHO	CC and WHO 1999	Hospital	2	50 g, 75 g/100 g	75	2000	3.8
Zhang et al., 2011	China	WHO	WHO 1999	Hospital	2	50 g, 75 g	4764	105,473	4.5
Zhang et al., 2015	China	IADPSG	IADPSG	Community	2	50 g, 75 g	1069	14,198	7.5
Zhu et al., 2017	China	CHINA MOH	CHINA MOH	Hospital	1	75 g	2987	14,986	19.9

## Abbreviations

ADA: American diabetes association
ADIPS: Australian diabetes in pregnancy society
CC: Carpenter-coustan
CDA: Canadian diabetes association
China MOH: China ministry of health
CI: Confidence interval
DIPSI: Diabetes in pregnancy study group of India
EASD: European association for the study of diabetes
GCT: Glucose challenge test
GDM: Gestational diabetes mellitus
IADPSG: International association of the diabetes and pregnancy study groups
ICD: International classification of diseases
JDS: Japan diabetes society
NDDG: National diabetes data group
OGTT: Oral glucose tolerance test
OR: Odds ratio
PCOS: Polycystic ovarian syndrome
PIH: Pregnancy induced hypertension
T2DM: Type 2 diabetes mellitus
WHO: World Health Organization
